# Exploration of differentially expressed mRNAs and miRNAs for pediatric acute myeloid leukemia

**DOI:** 10.3389/fgene.2022.865111

**Published:** 2022-09-06

**Authors:** Qing Wang, Chao Yue, Qin Liu, Xuchun Che

**Affiliations:** ^1^ Department of Clinical Laboratory, The General Hospital of Tianjin Medical University, Tianjin, China; ^2^ Department of Blood Transfusion, The Fifth Central Hospital of Tianjin, Tianjin, China; ^3^ Department of Clinical Laboratory, The Fifth Central Hospital of Tianjin, Tianjin, China; ^4^ Department of Immunology, Tianjin Key Laboratory of Cellular and Molecular Immunology, Key Laboratory of Educational Ministry of China, School of Basic Medical Sciences, Tianjin Medical University, Tianjin, China

**Keywords:** pediatric acute myeloid leukemia, differential gene analysis, hsa-miR-133, ZC3H15, BCLAF1

## Abstract

**Background:** To establish a comprehensive differential gene profile for pediatric acute myeloid leukemia patients (pAML) based on two independent databases and verify the differentially expressed genes using *in vitro* and *in vivo* analyses.

**Methods:** The mRNA and miRNA sequencing information of GSE2191 and GSE35320, clinically recruited pAML individuals, and human AML cell line (NB4 cells) were utilized in the study.

**Results:** Compared with the control sample, pAML patients demonstrated a total of 778 differentially expressed genes, including 565 upregulated genes and 213 downregulated genes. The genes including *ZC3H15*, *BCLAF1*, *PPIG*, *DNTTIP2*, *SRSF11*, *KTN1*, *UBE3A*, *PRPF40A*, *TMED5*, and *GNL2* were the top 10 potential hub genes. At the same time, 12 miRNAs demonstrated remarkable differential expressions in pAML individuals compared with control individuals, as five upregulated and seven downregulated miRNAs. The *hsa-miR-133*, *hsa-miR-181*, and *hsa-miR-195* were significantly downregulated. Building a miRNA–mRNA regulatory network, *hsa-miR-133* regulated *ZC3H15*, *BCLAF1*, *SRSF11*, *KTN1*, *PRPF40A*, and *GNL2*. Using the NB4 cell model, *hsa-miR-133* treatment inhibited cell proliferation capacity, which could be attenuated by a single mRNA transfection or a combination of *ZC3H15* and *BCLAF1*. At the same time, *hsa-miR-133* mimic treatment could significantly accelerate cell apoptosis in NB4 cells, which was also *ZC3H15*- and *BCLAF1*-dependent. The concentrations of ZC3H15 and BCLAF1 were investigated in peripheral blood using the ELISA method for the clinical control and pAML samples. In pAML samples, the expression levels of ZC3H15 and BCLAF1 were significantly enhanced (*p* < 0.01), regardless of the classification.

**Conclusion:** Collectively, this study hypothesized several promising candidates for pAML formation.

## Introduction

Acute myeloid leukemia (AML) is the most common malignancy of acute leukemia in humans, comprising approximately 80 percent of the cases (Short et al., 2018). Compared with adults, the incidence rate in children is relatively rare, but with disproportionate mortality (Elgarten and Aplenc, 2020). As recently reported, the pediatric acute myeloid leukemia (pAML) accounts for 25% of pediatric leukemia with a low overall survival ratio of 70% (Taga et al., 2016). Due to the decades of study, the treatments for pAML has been greatly improved by advances in the hematopoietic stem cell transplantation (HSCT) method, chemotherapy, supportive care, and optimal risk stratification (Tarlock and Meshinchi, 2015; Paulraj et al., 2019). Based on the various pAML subtypes, the clinical pAML procedures are conducted separately. For instance, children with *de novo* AML are administrated with a standard treatment, which includes four or five cycles of myelosuppressive chemotherapy with cytarabine and anthracyclines followed by a HSCT for a subset of patients. At the same time, children with acute promyelocytic leukemia (APL) are recommended with an all-trans retinoic acid (ATRA)–combined regimen. In contrast, myeloid leukemia children with Down syndrome (ML-DS) are generally converged with a less intensive regimen (Ribeiro, 2014).

The worldwide incidence of pAML is heterogeneous because of the variable prevalence of the risk factors. As reported, host factors such as age, race, and germline predisposition impact outcomes contribute to a large amount of pAML (Radhi et al., 2010; Shimada, 2017). In addition to the host factors, the elevated level of peripheral white blood cells (WBCs) has been also suggested as an unfavorable aspect for pAML (Woods et al., 1996). For adult AML, growing evidence based on retrospective assessments supports the association between the disease and the abnormal proliferation and differentiation of a clonal population of myeloid stem cells (De Kouchkovsky and Abdul-Hay, 2016). Except for the chromosomal rearrangements, the particular genetic mutations play a key role in the formation of AML, causing more than 97% of cases (Patel et al., 2012). Compared with adult AML, the pathological mechanism underlying pAML is still poorly understood and the molecular landscape behind pAML remains quite distinct (Bolouri et al., 2018). The treatments for pAML have evolved with years of work. If detected at an early stage, the pAML could be administrated with various choices of procedures. Unfortunately, for the majority of patients, pAML is diagnosed at a later stage so that the long-term prognosis remains unsatisfactory.

MicroRNAs (miRNAs) are emerging as a promising candidate for the molecular mechanism behind pAML, which attracts attention of many researchers. Previously, a study by Obulkasim et al. (2017)suggested that the biological subgroups of pAML were reflected by a common miRNA expression pattern, while the separate subtypes of pAML have distinct miRNA expression patterns. The miRNAs belong to a class of small endogenous RNAs that regulate gene expression post-transcriptionally and play a role in gene silencing and translation inhibition by binding to target genes. The miRNAs represent a highly conserved class of tissue-specific genes that have been found in all eukaryotic cells preserved across species since their discovery in 1993 (Vishnoi and Rani, 2017; Lu and Rothenberg, 2018). Generally speaking, they are short RNA molecules with 19–25 nucleotides in size. A single miRNA can target hundreds of mRNAs and influence the expression of many genes. The appropriate maintenance of miRNA expression is required for a balanced physiological environment.

Since the underlying molecular mechanism of pAML remains unclear, the disorder has brought great difficulty to clinical treatment. To address these issues, in this study, we used a combination of bioinformatics analysis and external clinical specimen experiments to establish differentially expressed gene profiling. Meanwhile, the potential signaling axis for pAML formation had also been explored. All of these promising outcomes enriched the precise early diagnosis of the disease, which provided tremendous help for the pAML study.

## Materials and methods

### Data source and differentially expressed gene analysis

The information on mRNA sequencing, miRNA sequencing as well as clinical data of children with AML were downloaded from the GEO database (GEO, https://www.ncbi.nlm.nih.gov/geo/).

The array chip numbering GSE2191 was established for pAML differentially expressed mRNA analysis. The database contains bone marrow samples from 54 pAML to 4 control individuals. All the specimens were used for analysis in the study. At the same time, the array chip numbering GSE35320 was utilized for pAML differentially expressed miRNA analysis. The database included the miRNA expression profiles of different AML subtypes from 102 pediatric patients in comparison to CD34+ cells from healthy donors and adult AML patients. In order to identify differentially expressed miRNAs for pAML, miRNA expression profiling of 110 pediatric AML patients was conducted for differentially expressed miRNA examination compared with 5 NB4, isotype control-–detected miRNAs.

The mRNA and miRNA expression analyses were performed by the genetic analysis group of HPS. Co (Tianjin, China). The information of selected samples was tested using the Affymetrix Human Genome U95 Version 2 Array platform.

For the original data measured by the chip, the RMA method was used to normalize first, and then the log2 logarithm of the normalized value was taken to generate the standardized data for downstream analysis. The limma package in R language (version:3.5.2) was developed to analyze the differentially expressed mRNAs as well miRNAs between different groups, taking the absolute value of the log-transformed differential expression multiple (Log2FC) > 1 and *p* < 0.05 as a standard for analysis.

Moreover, the final *p*-value was corrected using the Bonferroni method.

### Functional enrichment analysis

For the obtained differentially expressed genes, we used the “clusterProfiler” function package in R language for enrichment analysis of GO (including biological process, molecular function, and cellular component) and KEGG pathway. When *p*-value < 0.05, we considered the corresponding entries to be significantly enriched (Ritchie et al., 2015).

### Prediction of microRNA target genes

The target genes of miRNAs were predicted through the miRDB (http://mirdb.org/index.html, version 6.0) database (Yu et al., 2012). Meanwhile, the Cytoscape (https://cytoscape.org/, version 3.7.2) was performed to visualize the miRNA–mRNA regulatory network.

### Cell culture

The human AML cell line (NB4 cell line) was purchased from Procell Co., China (Cat. NO: CL-0676) and maintained in a RPMI-1640 medium with L-glutamine/10% fetal bovine serum/1X PSF.

The *hsa-miR-133* mimics and corresponding negative control (NC siRNA) were constructed by Genewiz Corporation Co. (Tianjin, China). Lipofectamine™ 3000 was used for plasmid transfection.

### Cell proliferation and apoptosis measurement

The cells were collected and measured for their proliferation capacity using the CCK-8 kit (Fisher, China). The absorbance was evaluated at 450 nm using the plate reader purchased from Thermo Fisher Scientific Co. At the same time, the cell apoptosis capacity was examined by flow cytometry after Annexin-V FITC/PI double staining. All experiments were conducted three times independently.

### Sample collection

This study was a retrospective study using clinical recruited pAML patients from General Hospital of Tianjin Medical University. The 110 pAML patients were selected based on the following inclusion criteria: 1) age from 0 to 18 years; 2) abnormal expression of leukemia, a significant increase in lymphocyte, varying degrees of anemia based on blood routine tests; 3) increased expression level of proliferating leukemia cells for bone marrow puncture; 4) positive for other auxiliary diagnostic schemes. The exclusion criteria: 1) presence of other malignancy or 2) other circulatory system disorders. A total of 28 control children were recruited in the control group, which were excluded from pAML, other malignancy or circulatory system disorders. Based on the standard of FAB classification (French–American–British classification), the pAML individuals were further sub-grouped into M0–M7 classifications (M0: 13; M1: 9; M2: 25; M3: 11; M4: 8; M5: 37; M6: 7 without M7).

This study was in line with the medical ethics standards, and approved by the hospital ethics committee. All treatment and testing were performed with informed consent of patients or their families. For the pAML group, there were 28 males and 20 females, and the average age was 9.92 ± 1.13 years. For the control group, there were 16 males and 12 females, and the average age was 9.64 ± 1.48 years. There was no significant difference between two groups (*p* > 0.05).

### ELISA analysis of hub genes

The protein concentrations of ZC3H15 and BCLAF1 were determined using the ELISA double antibody sandwich method from recruited individuals’ peripheral blood. The specific operation was carried out in strict accordance with the instructions of the kit (Abcam company, United States). The experimental results were repeated three times independently and were tested by statistical methods.

### Statistics

Excel 2022 was established for data analysis. The continuous variables were tested for normal distribution and continuous variables are presented by the mean ± standard deviation (x ± s). The student *t*-test was used for data comparison between two groups, with the *p* < 0.05 considered as a significant difference.

## Results

### Differentially expressed mRNA and miRNA analysis results

**FIGURE 1:**
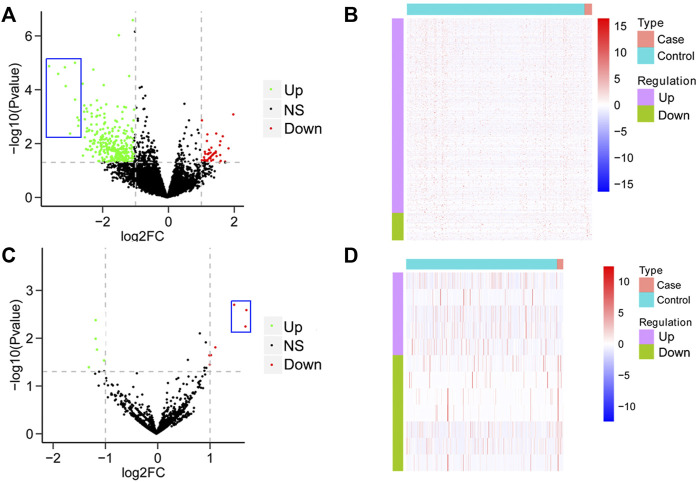
Analysis of differential mRNAs and miRNAs comparing pAML and control groups. **(A)** Volcano map of differentially expressed mRNAs between two groups. The horizontal axis represents the multiple differential expression (Log2FC), the vertical axis represents −log10 (FDR). The green dots indicate upregulated genes, and the red dots indicate downregulated genes, respectively. **(B)** Heat map of differentially expressed mRNAs. The horizontal axis represents the sample, vertical axis represents different genes. **(C)** Volcano map of differentially expressed miRNAs between two groups. **(D)** Heat map of differentially expressed miRNAs.

To establish a differential gene profiling for pAML, we first analyzed the mRNA information for pAML patients’ specimens on GSE2191. Compared with the control sample, pAML patients’ samples exhibited a total of 778 differentially expressed genes, including 565 upregulated genes and 213 downregulated genes (Figures 1A,B, Supplementary Table S1). The genes such as *ZC3H15*, *BCLAF1*, *PPIG*, *DNTTIP2*, *SRSF11*, *KTN1*, *UBE3A*, *PRPF40A*, *TMED5*, and *GNL2* were the top 10 potential hub genes (most significant difference among the two groups). It is interesting that all the primary hub genes were upregulated in pAML (indicated as blue rectangle in Figure 1A). At the same time, based on the investigation of array chip numbering GSE35320, 12 miRNAs demonstrated remarkable differential expressions in pAML individuals compared with control individuals, as five upregulated and seven downregulated miRNAs (Figures 1C,D; Supplementary Table S2). The *hsa-miR-133*, *hsa-miR-181*, and *hsa-miR-195* were significantly downregulated(indicated as blue rectangle in Figure 1C).

### GO and KEGG enrichment analysis results

**FIGURE 2:**
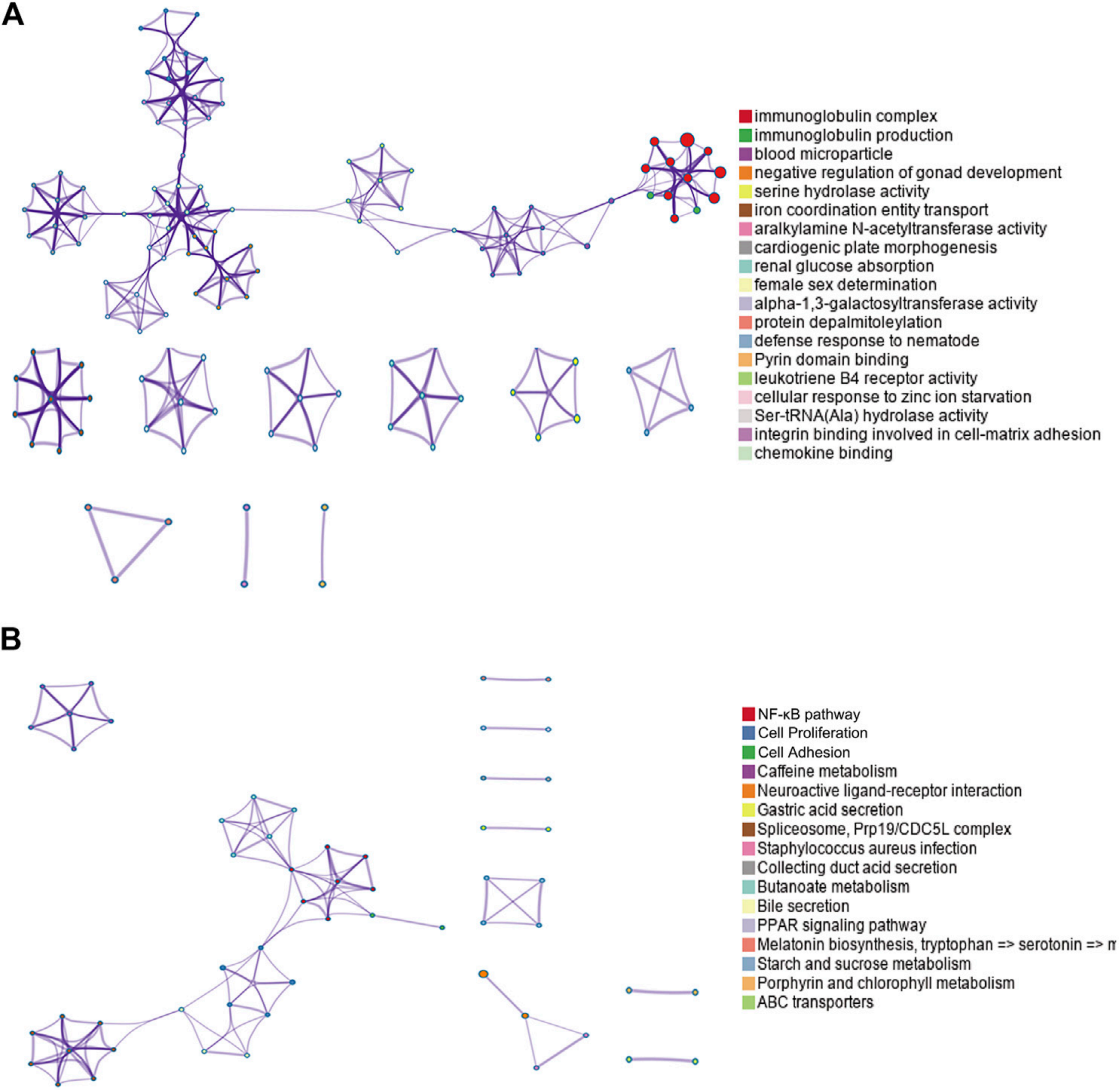
GO and KEGG enrichment results for differentially expressed genes. **(A)** Top GO term enrichment results with the largest number of genes. In the figure, the horizontal axis represents the number of enriched genes, and the vertical axis represents the name of each GO term, respectively. **(B)** Enrichment results of the KEGG pathways with the largest number of genes. The horizontal axis in the figure indicates the number of genes enriched, and the vertical axis indicates the name of each KEGG pathway, respectively.

By performing GO and KEGG enrichment analyses on these 778 differentially expressed genes, we found these differentially expressed genes were in GO terms related to various biological processes such as immunoglobulin complex and blood microparticles. (Figure 2A). In addition, the NF-κB pathway, cell proliferation, and cell adhesion–related signaling pathways were significantly enriched in KEGG pathway analysis (Figure 2B).

### miRNA–mRNA regulatory network

**FIGURE 3:**
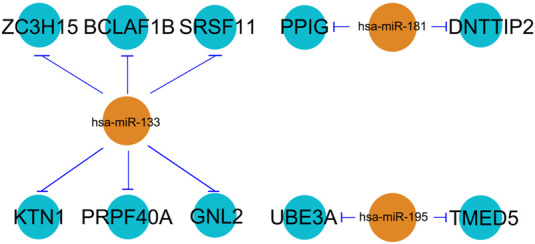
miRNA–mRNA regulatory network. The line type represents the interaction between miRNA and mRNA.

The three selected miRNAs (*hsa-miR-133*, *hsa-miR-181*, and *hsa-miR-195*) as well as 10 potential mRNAs (*ZC3H15*, *BCLAF1*, *PPIG*, *DNTTIP2*, *SRSF11*, *KTN1*, *UBE3A*, *PRPF40A*, *TMED5*, and *GNL2*) were further investigated for a regulatory network construction visualized using Cytoscape software. As shown in Figure 3, the three miRNAs were functionally associated with multiple hub genes. Among which, *hsa-miR-133* regulated the largest number of target genes (6), which were *ZC3H15*, *BCLAF1*, *SRSF11*, *KTN1*, *PRPF40A*, and *GNL2*. At the same time, *hsa-miR-181* interacted with two hub genes, which were *PPIG* and *DNTTIP2*. Meanwhile, the functions of *UBE3A* and *TMED5* were manipulated by *hsa-miR-195*.

### Verification of the differentially expressed genes using the *in vitro* cell model

**FIGURE 4:**
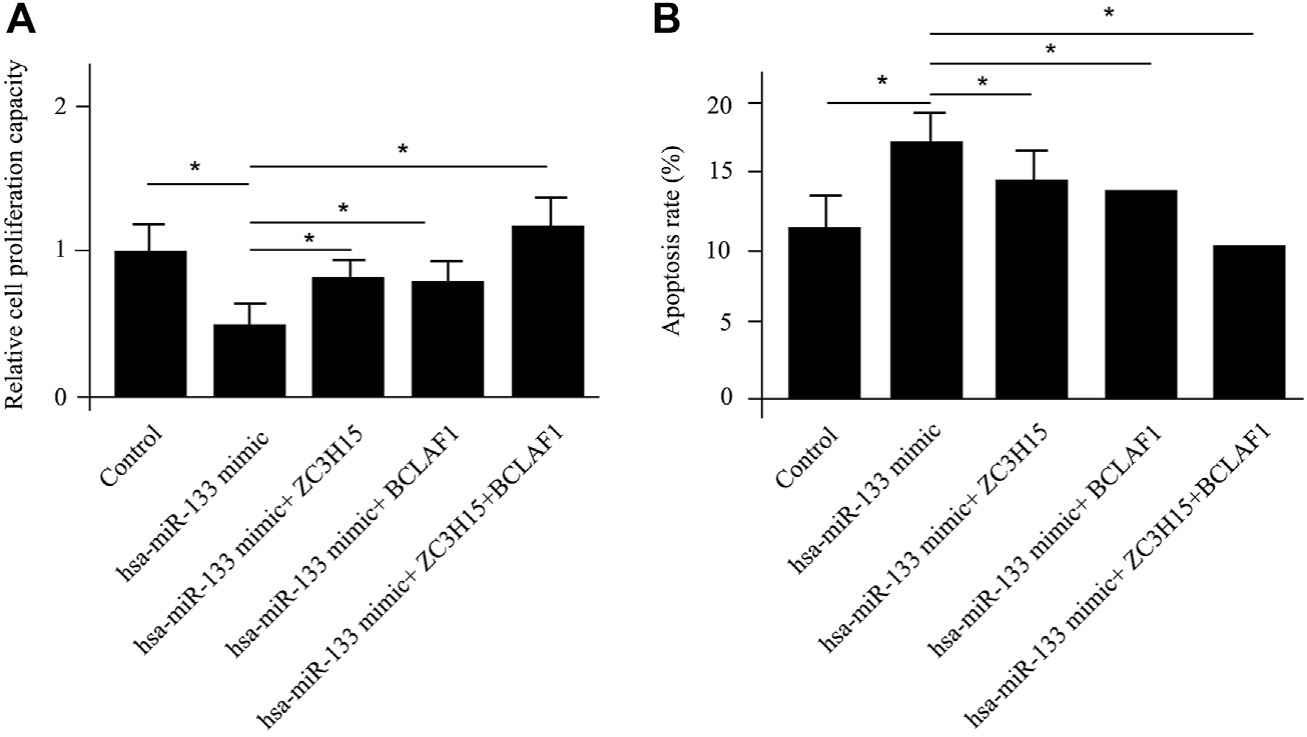
*In vitro* verification of differentially expressed mRNAs and miRNAs. The cell proliferation capacity **(A)** and cell apoptosis analysis **(B)** for NB4 cells with different treatments.

*ZC3H15* and *BCLAF1* were shown to be most significantly enhanced for pAML patients by differential gene analysis (Figure 1A). At the same time, both of the genes were demonstrated to be direct targets of *hsa-miR-133* by the miRNA–mRNA regulatory network results. Next, we sought to verify this using the *in vitro* cell model, which was the human AML cell line (NB4 cell line). The introduction of *hsa-miR-133* in NB4 cells obviously inhibited cell proliferation capacity, which could be attenuated by a single mRNA transfection or a combination of *ZC3H15* and *BCLAF1* (Figure 4A). Moreover, *hsa-miR-133* mimic treatment could significantly accelerate cell apoptosis in NB4 cells, which was also *ZC3H15*- and *BCLAF1*-dependent (Figure 4B). These outcomes suggested that the increased expressions of *ZC3H15* and *BCLAF1* and the decreased expression of *hsa-miR-133* were hallmarks for pAML. Meanwhile, *ZC3H15* and *BCLAF1* were direct targets for *hsa-miR-133* to modulate AML cellular functions.

### ZC3H15 and BCLAF1 were significantly differentially expressed for pAML *in vivo*

So far, *ZC3H15* and *BCLAF1* were suggested to be key regulatory factors for pAML progression, which were closely associated with cell proliferation and apoptosis. However, the pAML patients were sub-grouped into different classifications based on FAB criteria. The mRNA information of the array chip did not provide the inter-group special expression levels for the two key genes. In order to approve that the differential expression of mRNA was independent of FAB classification, we included pAML patients with different FAB classifications. To further verify the functions of hub genes in pAML patients, the protein expression levels of top two hub genes (*ZC3H15* and *BCLAF1*) were compared between clinical recruited control individuals and pAML individuals. The concentrations of ZC3H15 and BCLAF1 were investigated in peripheral blood using the ELISA method for the two groups.

For control samples, the protein concentration of ZC3H15 was 28.33 ± 5.51 ng/ml, ranging 21.3–35.4 ng/ml; while the protein concentration of BCLAF1 was 12.33 ± 3.39 ng/ml, ranging 9.2–19.4 ng/ml. On the other hand, in pAML samples (including all the FAB classifications), the expression levels of ZC3H15 and BCLAF1 were significantly enhanced (*p* < 0.01). The protein concentration of ZC3H15 was 45.68 ± 6.46 ng/ml, ranging 27.6–55.6 ng/ml; while the protein concentration of BCLAF1 was 22.17 ± 5.26 ng/ml, ranging 14.6–33.8 ng/ml. Furthermore, there were existing remarkable differences between control individuals and pAML patients of all the FAB groups (except for M7, which was not recruited in our study for the low sample size, as shown in Table 1). These outcomes were in line with the differential expression analysis results.

**TABLE 1:** Protein concentration of potential hub genes between control and different pAML groups.

Group	N	ZC3H15 (ng/ml, x ± s)	BCLAF1 (ng/ml, x ± s)
Control	28	28.33 ± 5.51*	12.33 ± 3.39*
pAML (total)	110	45.68 ± 6.46*	22.17 ± 5.26*
pAML (M0)	13	52.18 ± 6.35*	26.17 ± 5.08*
pAML (M1)	9	40.22 ± 5.73*	20.15 ± 4.29*
pAML (M2)	25	39.68 ± 5.33*	18.39 ± 4.21*
pAML (M3)	11	38.59 ± 6.12*	18.53 ± 3.88*
pAML (M4)	8	40.17 ± 6.31*	22.43 ± 5.40*
pAML (M5)	37	49.38 ± 7.02*	26.84 ± 6.25*
pAML (M6)	7	48.11 ± 7.11*	24.21 ± 6.32*

*as an indication of *p* < 0.05 compared with the control group.

## Discussion

Even with years of hard work for the breakthroughs in pAML, the prevention, early screening, diagnosis, treatments, and overall outcomes are still inferior, highlighting the need for improved targeted therapies. In this study, we comprehensively compared differentially expressed mRNAs as well as miRNAs between pAML and control groups based on two independent databases. In this integrated study, the 10 mRNAs and three miRNAs were hypothesized as key regulators for pAML formation. Previously, more and more attention had been paid to the interaction between the signal transducer and activator of transcription 3 (STAT3) and AML in adults. Increases in cytokine ligands, such as IL-6, trigger intracellular tyrosine phosphorylation of STAT3, which is seen in up to 50% of AML cases and signifies a worse prognosis (Schuringa et al., 2000). In addition to STAT3, NPM1 and CEBPA confer to notable class II mutations while DNA-methylation–related genes DNMT3A, TET2, and IDH-1 and IDH-2 contribute to the class II mutation group for AML, which are found in about 27% and more than 40% of AML cases, respectively (Takahashi, 2011; Klein et al., 2018). However, the majority of these mutations were not significantly expressed in the pAML group specimens from our analysis, which might reflect a tremendous difference between pediatrics and adults for AML initiation as well as development. The DNA methylation events in somatic mutations are suggested to be highly prevalent in adults. Inversely, the structural alterations in methyltransferase genes are more prevalent in young children, but rarer or even absent in adults. These may explain the differences for the AML between children and adults considering differentially expressed gene profiles.

Previously, the connection between miRNA and leukemogenesis was built on the cell proliferation as well as cell adhesion regulation by particular miRNA families (Mott et al., 2007; Volinia et al., 2010). These are in dire need of better understanding of the functions of miRNA in pAML since most of the work focuses on the adult AML (Danen-van Oorschot et al., 2012). A study by Zhang et al. (2009) demonstrated the miR-expression differences between FAB-M1, FAB-M2, and FAB-M3 groups in pAML. Another study suggested the specific expression pattern of miR-99a, miR-125b, and let-7c in pAML and the upregulations of these factors stimulated leukemogenesis by switching the balance between TGF-ß and Wnt signaling pathways (Bonifant et al., 2018). In a large cohort, Obulkasim and his colleagues analyzed 665 miRNAs on 165 pediatric AML samples, which provided 14 key clusters for the differentially expressed miRNAs (Lu and Rothenberg, 2018). In this study, they claimed pAML samples with MLL rearrangements were classified with 89% accuracy using 37 miRNA expression signatures; 14 miRNAs were highly expressed and 23 were lowly expressed compared to the samples without MLL rearrangements. In consistent with our findings here, *hsa-miR-133*, *hsa-miR-181*, and *hsa-miR-195* were strikingly repressed in the pAML samples. Moreover, they supposed that *hsa-miR-181* and *hsa-miR-195* were functional as tumor suppressors while *hsa-miR-133* was only used to classify MLL-rearranged samples in pAML.

Except for miRNA analysis, we also hypothesized the key mRNAs for pAML, including *ZC3H15*, *BCLAF1*, *PPIG*, *DNTTIP2*, *SRSF11*, *KTN1*, *UBE3A*, *PRPF40A*, *TMED5*, and *GNL2.* ZC3H15 represents zinc finger CCCH-type containing 15, which is an immediate early erythropoietin response gene. Using the co-immunoprecipitation method, it can be found that ZC3H15 is functional via the signaling adapter protein tumor necrosis factor receptor–associated factor 2 (TRAF-2) with the NF-κB pathway for AML formation (Capalbo et al., 2013). Here, in this study, we also observed the key roles of ZC3H15 for pAML. Meanwhile, the NF-κB pathway was suggested as the primary signaling pathway by KEGG analysis (as shown in Figure 2). Taken these together, ZC3H15 may be a central factor for the pAML process through the NF-κB signaling pathway, raising an interesting direction for future pathological mechanism studies underlying pAML. BCLAF1 was originally initiated as a key of apoptosis and repressor of transcription, which is associated with antiapoptotic members of the Bcl2 family for various developmental processes such as T-cell activation and so on (Sarras et al., 2010). SRSF11 was previously demonstrated as a novel TERC-binding protein, which localizes to nuclear speckles and associates with active telomerase enzyme for cell cycle manipulation (Lee et al., 2015). Based on its function as a nuclear speckle–targeting factor essential for telomerase association with telomeres, SRSF11 has been deeply explored in cancer research. UBE3A is a dual-function protein, which consists of ubiquitin ligase as well as transcriptional co-activator function and has been shown to play a fundamental role in the modulation of synaptic function and plasticity (Vatsa and Jana, 2018). Except for these, the association between selected hub genes from this study and pAML has not been fully explored, which calls for a great point for future studies. Since *ZC3H15* and *BCLAF1* were suggested as two primary hub genes for pAML. We further investigated the expression level of them between control and pAML groups. Initially, we analyzed the expression in the cell line and observed the distinct expression pattern for both of them (data not shown). We preferred to utilize peripheral blood samples since pAML refers to a blood disease. Using clinical specimens, both ZC3H15 and BCLAF1 displayed a remarkable increased expression level in the pAML compared with the control group (as shown in Table 1), which confirmed the functions of these genes in clinical verification. It is worth noting that all the top hub genes showed increased expressions in the pAML group, which could be explained by the downregulations of top three miRNAs (as shown in Figures 1, 3). All of these deserve further investigation.

To conclude, in the light of unclear pathological mechanisms as well as missing potential biomarkers for pAML, we systematically generated a differential expression profile of mRNAs and miRNAs and provided several potential miRNA-dependent signaling axes for pAML patients. All the work here offered novel insights for the future pAML research.

## Data Availability

The original contributions presented in the study are included in the article/Supplementary Material; further inquiries can be directed to the corresponding authors.
